# Flow Characteristics of Heat and Mass for Nanofluid under Different Operating Temperatures over Wedge and Plate

**DOI:** 10.3390/mi13122080

**Published:** 2022-11-26

**Authors:** Muhammad Rizwan, Mohsan Hassan, Muhammad Imran Asjad, ElSayed M. Tag-ElDin

**Affiliations:** 1 Department of Mathematics, COMSATS University Islamabad, Lahore 54000, Pakistan; 2 Department of Mathematics, University of Management and Technology, Lahore 54770, Pakistan; 3 Faculty of Engineering and Technology, Future University in Egypt, New Cairo 11835, Egypt

**Keywords:** nanofluid, flow characteristics, temperature-dependent thermophysical properties, metallic oxides and carbon-nanostructure

## Abstract

Background and Purpose: Nanofluids are a new class of heat transfer fluids that are used for different heat transfer applications. The transport characteristics of these fluids not only depend upon flow conditions but also strongly depend on operating temperature. In respect of these facts, the properties of these fluids are modified to measure the temperature effects and used in the governing equations to see the heat and mass flow behavior. Design of Model: Consider the nanofluids which are synthesized by dispersing metallic oxides (SiO_2_, Al_2_O_3_), carbon nanostructures (PEG-TGr, PEG-GnP), and nanoparticles in deionized water (DIW), with (0.025–0.1%) particle concentration over (30–50 °C) temperature range. The thermophysical properties of these fluids are modeled theoretically with the help of experimental data as a function of a temperature and volume fraction. These models are further used in transport equations for fluid flow over both wedge and plate. To get the solution, the equations are simplified in the shape of ordinary differential equations by applying the boundary layer and similarity transformations and then solved by the RK method. Results: The solution of the governing equation is found in the form of velocity and temperature expressions for both geometries and displayed graphically for discussion. Moreover, momentum and thermal boundary layer thicknesses, displacement, momentum thicknesses, the coefficient of skin friction, and Nusselt number are calculated numerically in tabular form. Finding: The maximum reduction and enhancement in velocity and temperature profile is found in the case of flow over the plate as compared to the wedge. The boundary layer parameters are increased in the case of flow over the plate than the wedge.

## 1. Introduction

The nanofluids are engineered colloidal suspensions of nano-sized particles in conventional fluids (water, EG, or oil) [1,2,3,4,5,6,7,8,9,10]. Mostly, nanoparticles of carbides, oxides, and metals are used to synthesize the nanofluids. The nanofluids are usually used as coolants in various heat transfer equipment, such as electronic cooling systems, heat exchangers, radiation, etc., due to their improved thermophysical properties [11,12,13,14,15,16,17,18].

Many investigations have been carried out to see the behavior of thermophysical properties of nanofluids for different applications by using different types of nanoparticles [19,20,21,22,23]. In the list of thermophysical properties, viscosity plays an important role in the transport of mass and convective heat transfer. The viscosity of nanofluid is affected not only by shear rate but also by operating temperature, nanoparticle concentration, type of nanoparticles and their sizes, etc. Numerous studies have been conducted on the behavior of viscosity of nanofluids. Moghaddam et al. [24] studied the viscosity of graphene/glycerol nanofluids at a 6.32 shear rate, 20 °C temperature, and different particle concentrations. It increases by increasing nanoparticle concentration and decreases by enhancing temperature. Chen et al. [25] described the rheological properties of TiO2/EG nanofluids. The results exhibit the Newtonian at a 0.5–10^4^ shear rate and found that viscosity is independent of the temperature. Rashin and Hemalatha [26] investigated the viscosity of CuO/coconut oil nanofluids. Their experiments showed the non-Newtonian behavior at a low shear rate (0–2.5%) concentration under different temperatures. Khedkar et al. [27] studied the viscosity of Fe_3_O_4_/paraffin at 0.01–0.1% concentration. Their experimental results showed that the viscosity is enhanced by increasing nanoparticle concentration whereas it shows Newtonian behavior at a high shear rate and non-Newtonian at a lower. Halelfadl et al. [28] studied the viscosity of CNT/water nanofluids at a high shear rate under different temperature conditions. The results showed that the nanofluids performed a non-Newtonian behavior at high nanoparticle concentration and Newtonian at lower nanoparticle concentration. Later, Chen et al. [29] studied the rheological properties of TiO_2_/EG nanofluids at different nanoparticle concentrations and temperatures. The nanofluids show a non-Newtonian property at 2% particle concentration under different temperatures. Numburu et al. [30] investigated the rhetorical property of SiO_2_/EG and SiO_2_/water nanofluids at −35–50 °C temperature. It is found that the nanofluid exhibited Newtonian properties at high temperatures and non-Newtonian properties at low temperatures. Kulkarni et al. [31] reported the viscosity of Al_2_O_3_/EG, CuO/EG, and SiO_2_/EG nanofluids under −35–50 °C temperature ranges. It is reported that viscosity reduces exponentially by increasing temperature. Yu et al. [32] observed the effects of the viscosity of ZnO/EG nanofluids. The results detected Newtonian behaviors at low particle concentrations and non-Newtonian behaviors at higher particle concentrations under different temperature conditions. 

In the literature related to nanofluids, the behavior of thermal conductivity is investigated widely due to heat transfer’s applications, and found that the behavior of conduction depends on various factors such as temperature, nanoparticle shape, size, and type [33,34]. Teng et al. [35] investigated the impact of a particle’s size and temperature on the thermal conductivity of Al_2_O_3_/H_2_O nanofluids. The results exhibit that the thermal conductivity is increased with increasing nanoparticles concentration and temperature. Chandrasekar et al. [36] observed that the thermal conductivity of Al_2_O_3_/water nanofluids increased by increasing nanoparticle concentration under room temperature. Sundar et al. [37] predicted the behavior of thermal conductivity and viscosity of Al_2_O_3_/EG-Water nanofluids on different particle concentrations (0.3–1.5%) at temperatures range (20–60 °C). The results specified that the thermal conductivity of nanofluids improves with increasing nanoparticle concentrations and temperatures. Mahbubul et al. [38] studied the behavior of the thermal conductivity of Al_2_O_3_/R141b nano-refrigerant and found an enhancement in thermal conduction by increasing nanoparticle concentration and temperature. Mostafizur et al. [39] investigated the thermal conductivities of SiO_2_/methanol, Al_2_O_3_/methanol, and TiO_2_/methanol nanofluids. It was concluded that the thermal conductivity is increased for all nanofluids but found higher for Al_2_O_3_/methanol nanofluids as compared to the other two nanofluids. Das et al. [40] studied the thermal conductivity in different ranges of temperature for five distinct nanofluids which are prepared by dispersion of SiO_2_, Al_2_O_3_, TiO_2_, CuO, and ZnO nanoparticles in propylene glycol-water. The improvement in thermal conductivity of all nanofluids by enhancing temperature and nanoparticle concentration is found. Murshed et al. [41] investigated the thermal conductivity of TiO_2_/DI H_2_O nanofluids. Their experiments show the enhancement in thermal conductivity by increasing particle concentration (0.5–5%) at room temperature. Duangthongsuk and Wong wises [42] detected the behavior of thermal conductivity of TiO_2_/H_2_O nanofluids. The thermal conductivity of nanofluids increased by nanoparticle concentration as well as increased temperature.

In the above studies, it is found that the nature of fluid whether it is Newtonian or non-Newtonian depends on the behavior of viscosity. The behavior of viscosity is not only changed by nanoparticles but also depends on operating temperature. Similarly, the thermal conductivity of the nanofluid not only increased by nanoparticle concentration but also increased by increasing temperature. Keeping in mind these facts, the rheological properties of four different nanofluids such as SiO_2_/DIW, Al_2_O_3_/DIW, PEG-TGr/DIW, and PEG-GnP/DIW are modeled as a function of nanoparticle concentration and operating temperature in the current study. For modeling, experimental data is picked at 0.025%, 0.05%, 0.075%, and 0.1% nanoparticle concentration under 30 °C, 40 °C, and 50 °C temperature range [1]. Further, these models are used in transport equations to see the boundary layer flow over two different geometries such as wedge and plate. The whole investigation is divided into different sections. After introductions in Section 1, the mathematical models are established based on experimental data to discuss the thermophysical properties and parameters of schematic nanofluids in the form of graphs and tables respectively in Section 2. In Section 3, the mathematical problem for flow is developed by using continuity, momentum, and energy equations. In Section 4, physical parameters such as momentum and thermal boundary layers thickness, momentum and displacement thicknesses, coefficient of skin friction, and Nusselt number are modeled. The numerical solution of the problem is obtained using the RK method and gets the solutions in the form of velocity and temperature functions. In next Section 6, attained results are displayed in graphical and tabular form for discussion. In last Section 7, the significant outcomes are concluded.

## 2. Nanofluid Modeling

### 2.1. Viscosity Model

Consider the following viscosity model as

(1) $$\mu \left(T,\dot{\gamma }\right)=\mu_{1}\left(T\right)\mu_{2}\left(\dot{\gamma }\right),$$

Here, T is the temperature, $\dot{\gamma }$ is the shear rate, $\mu_{1}\left(T\right)$ and $\mu_{2}\left(\dot{\gamma }\right)$ are temperature and shear rate depended functions. The $\mu_{1}\left(T\right)$ is taken as exponential form whereas power law model is taken for $\mu_{2}\left(\dot{\gamma }\right)$ which are defined as

(2) $$\mu =\mu_{nf}{\left|\dot{\gamma }\right|}^{n-1}e^{-C_{1}\left(T-T_{\infty }\right)}.$$

**Figure 1:**
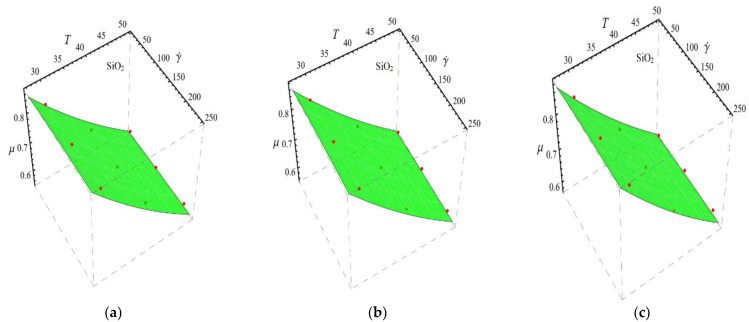
Fitting the Equation (2) for experimental data of SiO_2_/DIW nanofluid at different nanoparticle volume fractions (**a**) ϕ=0.025%, (**b**) ϕ=0.05% and (**c**) ϕ=0.075%.

**Figure 2:**
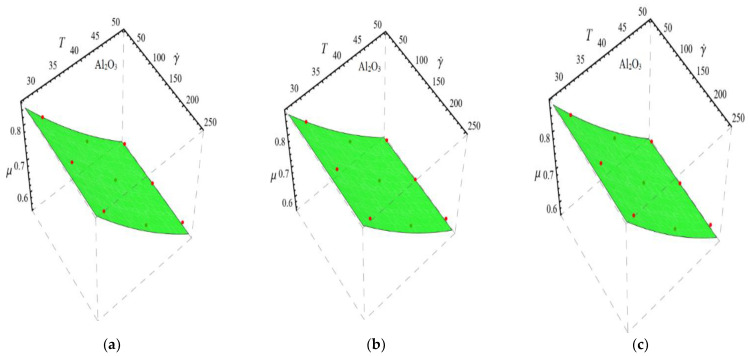
Fitting the Equation (2) for experimental data of Al_2_O_3_/DIW nanofluid at different nanoparticle volume fractions (**a**) ϕ=0.025%, (**b**) ϕ=0.05% and (**c**) ϕ=0.075%.

**Figure 3:**
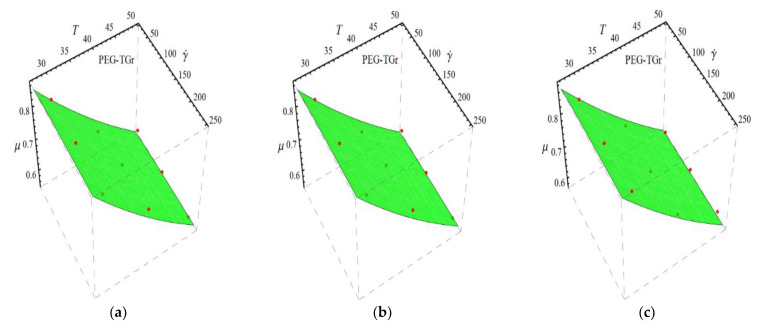
Fitting the Equation (2) for experimental data of PEG-TGr/DIW nanofluid at different nanoparticle volume fractions (**a**) ϕ=0.025%, (**b**) ϕ=0.05% and (**c**) ϕ=0.075%.

**Figure 4:**
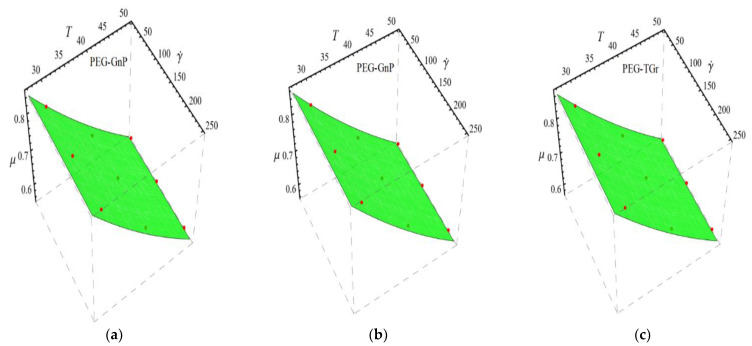
Fitting the Equation (2) for experimental data of PEG-GnP/DIW nanofluid at different nanoparticle volume fractions (**a**) ϕ=0.025%, (**b**) ϕ=0.05% and (**c**) ϕ=0.075%.

Here n, $\mu_{nf}$ and $C_{1}$ are curve fitting parameters. The numerical values of these parameters are obtained by fitting the Equation (2) to experimental data [1]. The best-fitting results are presented in Figure 1, Figure 2, Figure 3 and Figure 4. In these figures, dots represent the experimental data [1] and graph illustrates the Equation (2).

In respect of Figure 1, Figure 2, Figure 3 and Figure 4, the values of curve fitting parameters $\mu_{nf},$n and $C_{1}$ are listed in Table 1.

Furthermore, the above values are fitted into a second-degree polynomial Equation (3) as expressed as

(3) $$P\left(\phi \right)=a+b\phi \;+c\phi^{2}.$$

**Figure 5:**
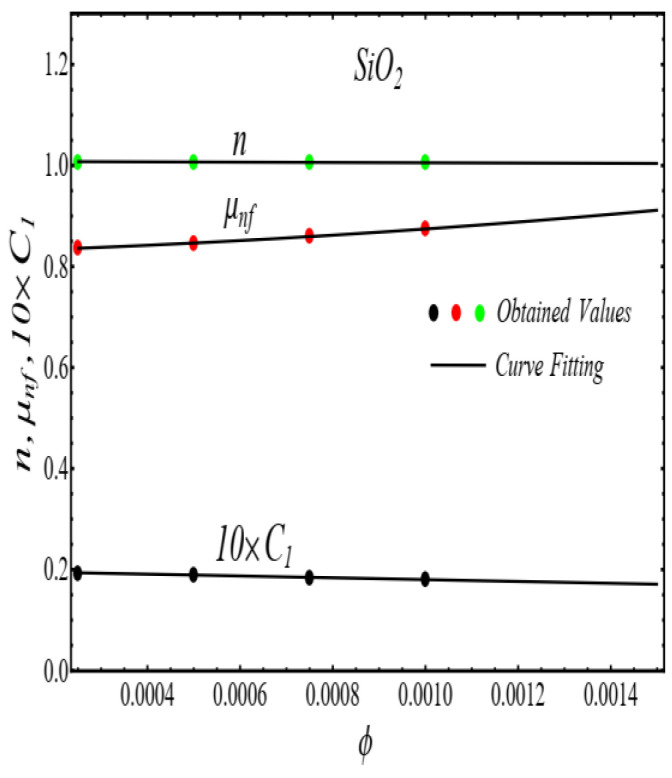
Fitting the Equation (3) for data in Table 1.

**Figure 6:**
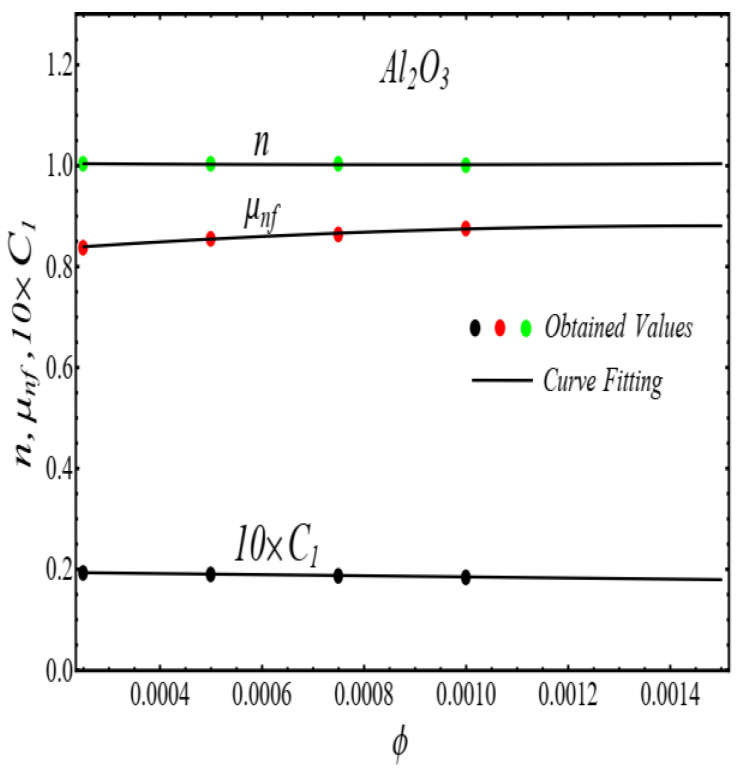
Fitting the Equation (3) for data in Table 1.

**Figure 7:**
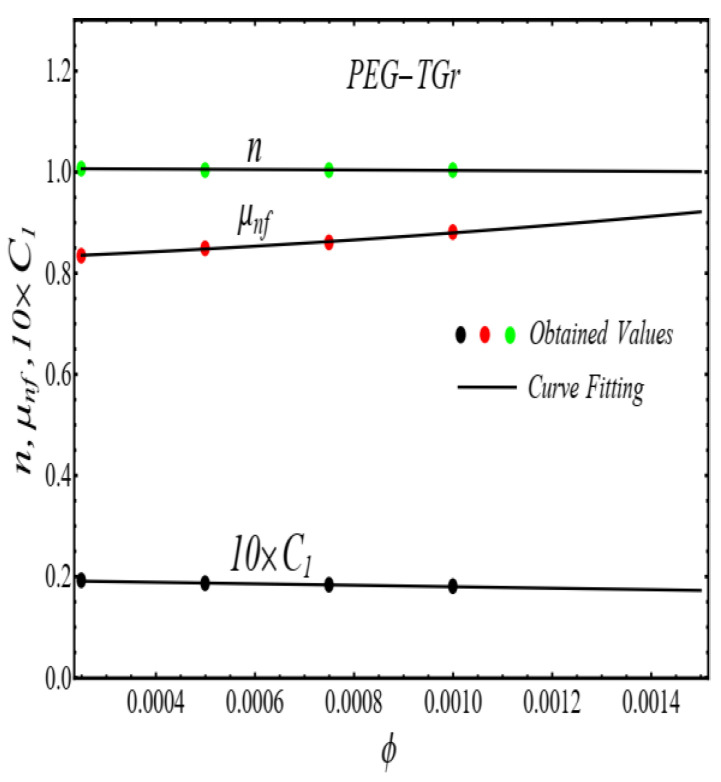
Curve fitting the Equation (3) for data in Table 1.

**Figure 8:**
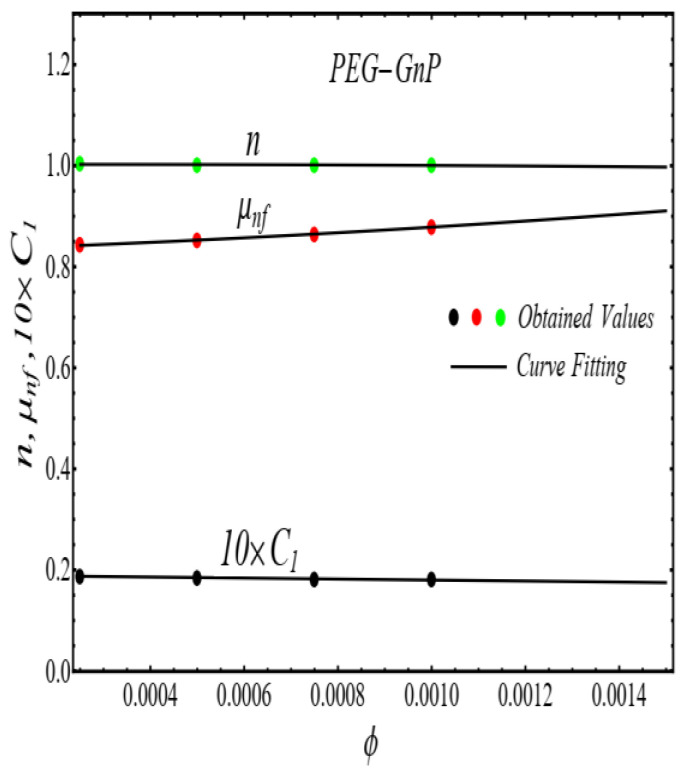
Curve fitting the Equation (4) for specified data in Table 1.

The results of curve fitting are displayed in Figure 5, Figure 6, Figure 7 and Figure 8 and the values of coefficients of Equation (3) are written in Table 2.

### 2.2. Thermal Conductivity Model

**Figure 9:**
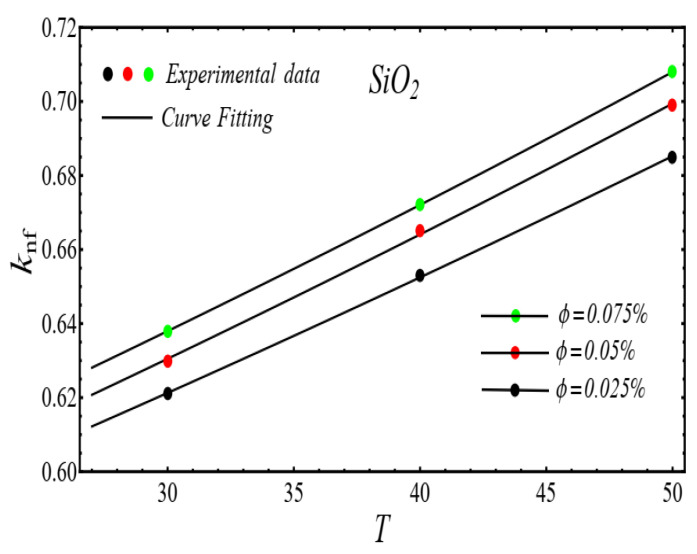
Fitting the Equation (4) for experimental data of SiO_2_/DIW nanofluid at different nanoparticle volume fractions.

**Figure 10:**
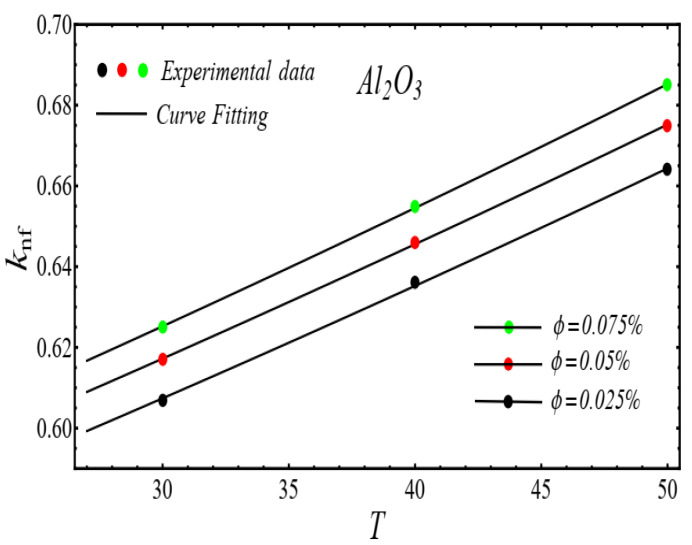
Fitting the Equation (4) for experimental data of Al_2_O_3_/DIW nanofluid at different nanoparticle volume fractions.

**Figure 11:**
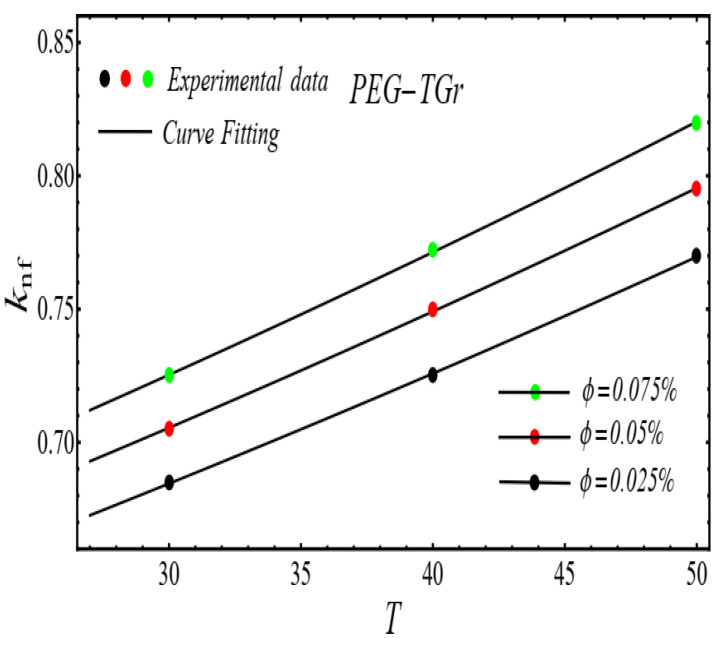
Fitting the Equation (4) for experimental data of PEG-TGr/DIW nanofluid at different nanoparticle volume fractions.

**Figure 12:**
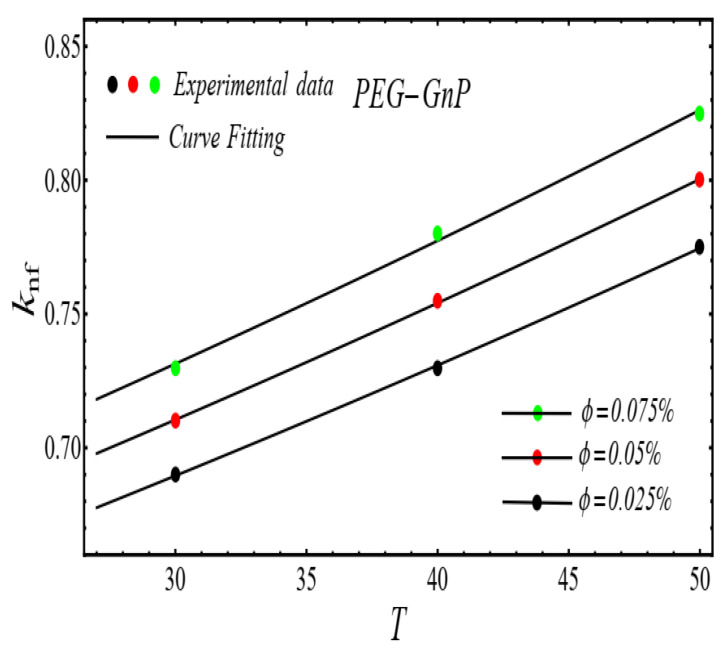
Fitting the Equation (4) for experimental data of PEG-GnP/DIW nanofluid at different nanoparticle volume fractions.

Consider the thermal conductivity model on the pattern of Equation (1) as

(4) $$k_{nf}=ke^{C_{2}\left(T-T_{\infty }\right)},$$

where, k and $C_{2}$ are curve fitting parameters. The values of these parameters are obtained by fitting the Equation (4) for experimental data [1]. The results of curving fitting are displayed in Figure 9, Figure 10, Figure 11 and Figure 12 and the values of curve fitting parameters are displayed in Table 3.

**Figure 13:**
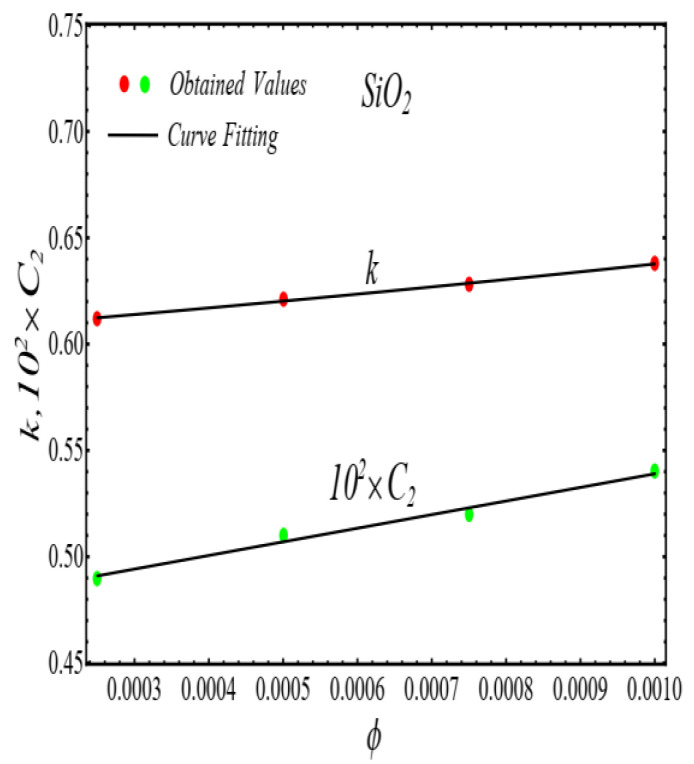
Fitting of the Equation (4) for parameters listed in Table 3.

**Figure 14:**
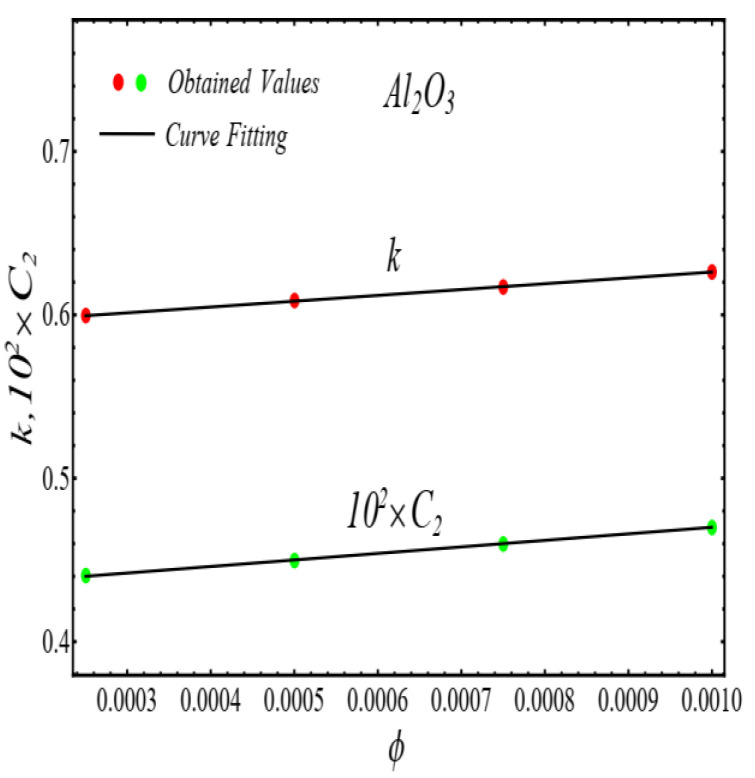
Fitting of the Equation (4) for parameters listed in Table 3.

**Figure 15:**
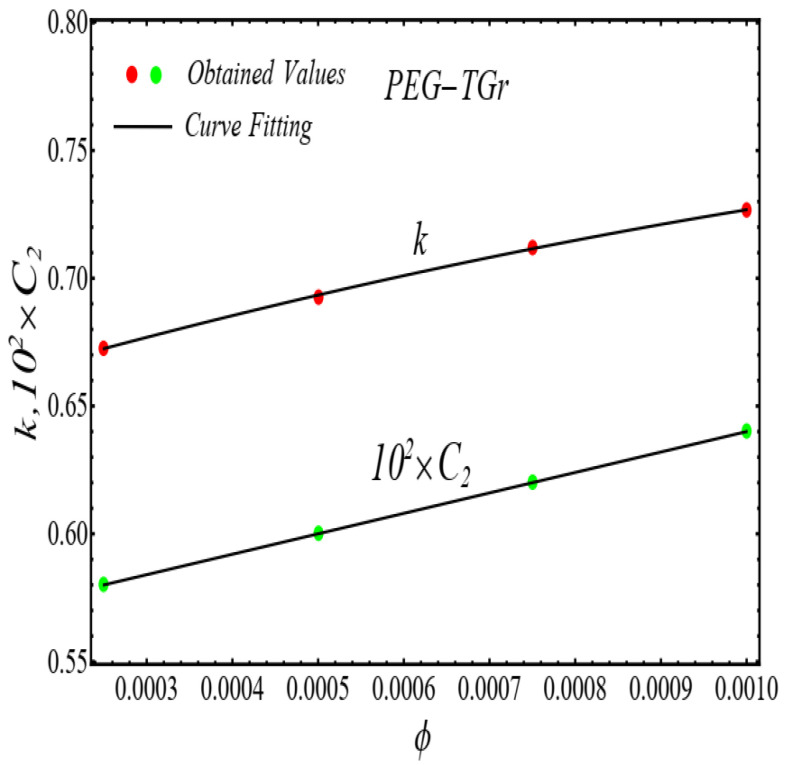
Fitting of the Equation (4) for parameters listed in Table 3.

**Figure 16:**
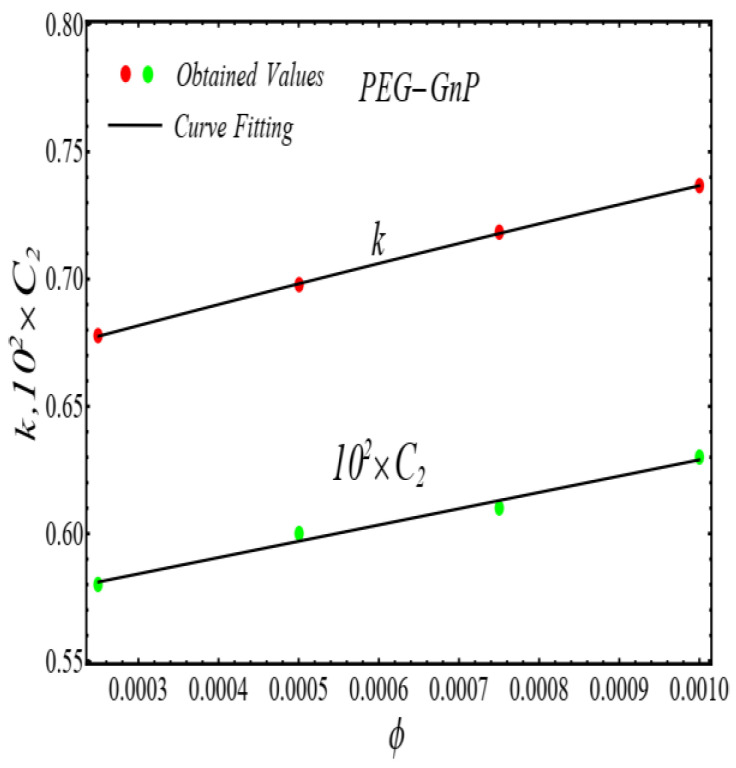
Fitting of the Equation (4) for parameters listed in Table 3.

Moreover, the values of the parameters in Table 3 are fitted into Equation (3) and their results are presented in Figure 13, Figure 14, Figure 15 and Figure 16.

### 2.3. Density and Heat Capacity Models

The co-relation models for density $\left(\rho_{nf}\right)$ and heat capacity ${\left(C_{p}\right)}_{nf}$ are developed by fitting the polynomial of 1st-degree Equation (5) for experimental data [1]

(5) $$P\left(\phi \right)=a+b\phi$$

**Figure 17:**
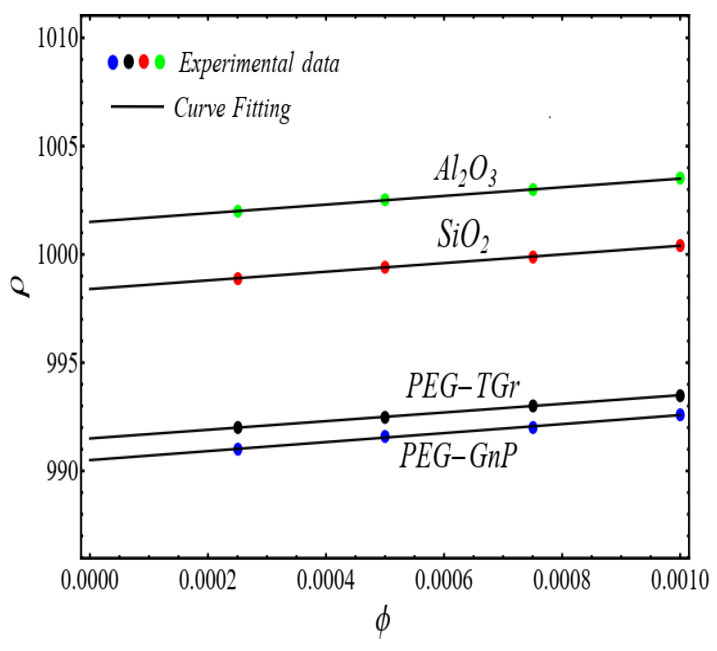
Fitting the Equation (5) for experimental data of schematic nanofluids.

**Figure 18:**
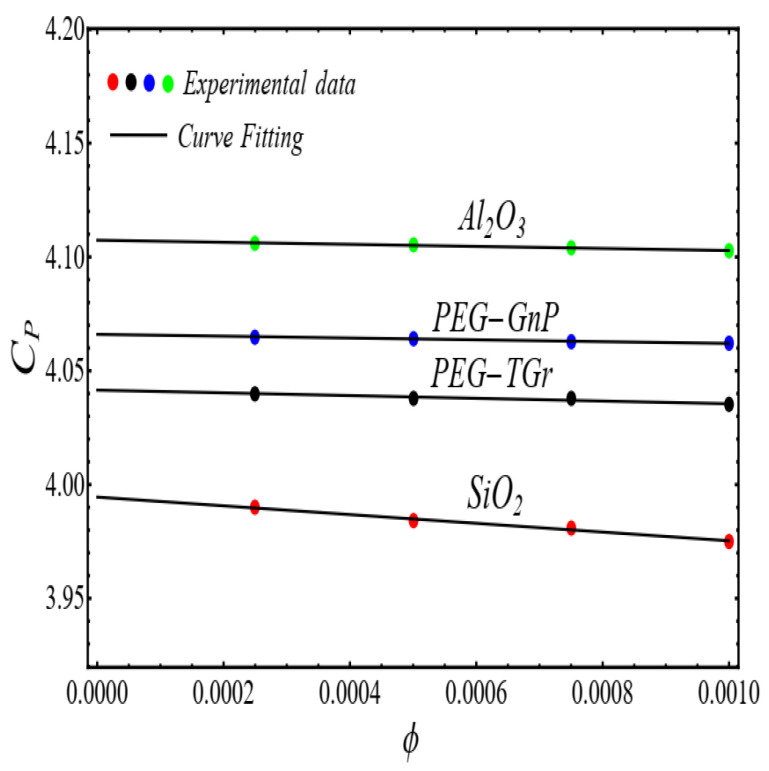
Fitting the Equation (5) for experimental data of schematic nanofluids.

The results of curve fitting are displayed in Figure 17 and Figure 18.

In view of Figure 17 and Figure 18, the values of the co-efficient of Equation (5) are displayed in Table 4.

## 3. Heat and Mass Flow Modeling

**Figure 19:**
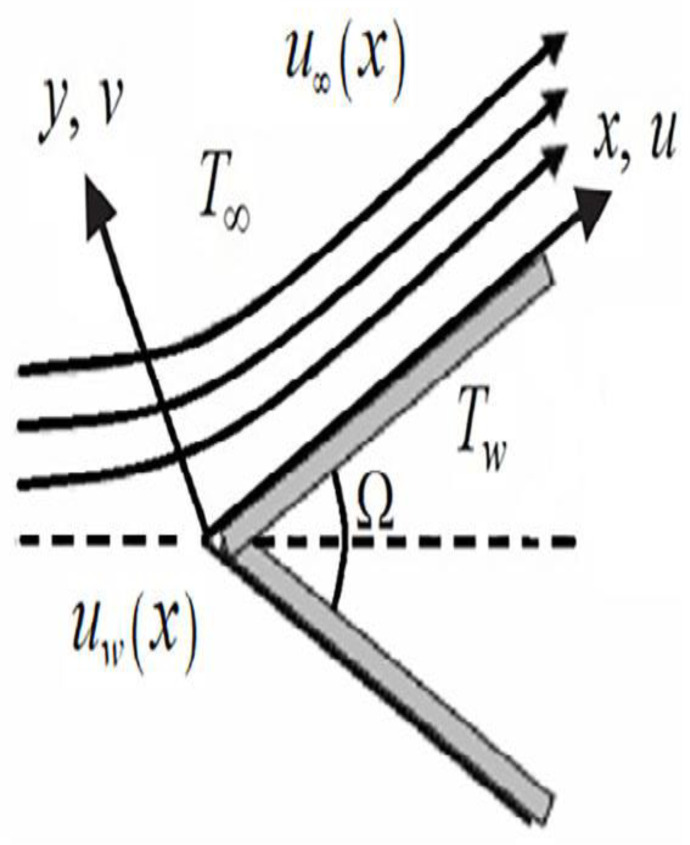
Flow Structure over geometry.

Consider the steady state and an incompressible boundary layer fluid flow propagating over two different geometries (Plate and Wedge). The fluid at the wall flowed with $u_{w}\left(x\right)=bx^{m}$ velocity and flowed with $u_{e}\left(x\right)=cx^{m}$ velocity in the free stream region as seen in Figure 19. The relationship between the Falkner-Skan power law parameter $\left(m\right)$ and the wedge’s angle β=Ω/π is stated as

(6) $$\beta =\frac{2m}{m+1}$$

Geometry exhibited plate-shaped when m=0 and wedge when m>0. The temperature at the wall and away from the wall is maintained with constant $T_{w}$ and $T_{\infty }$ i.e., $\left(T_{w}>T_{\infty }\right)$ respectively.

Under the boundary layer approximation, the continuity, momentum, and energy equations can be written as

(7) $$u_{x}+v_{y}=0$$

(8) $$\rho_{nf}\left(u\;u_{x}+v\;u_{y}\right)=\rho_{f}u_{e}\partial_{x}\left(u_{e}\right)+\partial_{y}\left(\mu \;u_{y}\right)-{\left(\rho \beta \right)}_{nf}g\sin\left(\frac{\Omega }{2}\right)\left(T_{w}-T_{\infty }\right)$$

(9) $${\left(\rho C_{p}\right)}_{nf}\left(u\;T_{x}+v\;T_{y}\right)=\partial_{y}(k\;T_{y})+\mu_{nf}{\left(u_{y}\right)}^{2}$$

with the boundary conditions

(10) $$\left.\begin{array}{l} u\left(x,0\right)=-u_{w}\left(x\right),\;\;v\left(x,0\right)=0,\;T\left(x,0\right)=T_{w}\;\\ u\left(x,\infty \right)=u_{e}\left(x\right),\;T\left(x,\infty \right)=T_{\infty }\; \end{array}\right\}$$

For simplicity, introduced the similarity transformations [43] as

(11) η=yxRex12, ψ=uexRex−12fη, θη=T−T∞Tw−T∞,u=∂ψ∂y,v=−∂ψ∂x.

After the substitution of Equation (11) into Equation (6)–(8), we obtain the following non-dimensional equations

(12) $$\frac{\mu_{nf}}{\mu_{f}}\left(f^{\prime \prime \prime }-Af^{\prime \prime }\theta^{\prime }\right)e^{-AT}+\frac{\rho_{nf}}{\rho_{f}}\left(\left(\frac{m+1}{2}\right)ff^{\prime \prime }-m{\left(f^{\prime }\right)}^{2}\right)+m+\omega \frac{{\left(\rho \beta \right)}_{nf}}{{\left(\rho \beta \right)}_{f}}\sin\left(\frac{\Omega }{2}\right)\theta =0$$

(13) $$\frac{k_{nf}}{k_{f}}\left(B{\left(\theta^{\prime }\right)}^{2}+\theta^{\prime \prime }\right)e^{BT}+\mathrm{Pr}\frac{{\left(\rho C_{p}\right)}_{nf}}{{\left(\rho C_{p}\right)}_{f}}\left(\frac{m+1}{2}\right)f\theta^{\prime }+\mathrm{Pr}.Ec.\frac{\mu_{nf}}{\mu_{f}}{\left(f^{\prime \prime }\right)}^{2}=0$$

(14) $$\left.\begin{array}{c} f\left(0\right)=\;0,\;f^{\prime }\left(0\right)=\lambda ,\;\theta \left(0\right)=1,\;\\ \theta \left(\infty \right)=0,\;f^{\prime }\left(\infty \right)=1, \end{array}\right\}$$

Here, $\mathrm{Pr}=\frac{\mu_{f}c_{p}}{k_{f}}$ is Prandtl Number, Rex=ρfuexμf is Reynold number, ω=GrxRex2 is the mixed convection parameter, $Gr_{x}=\frac{\beta_{f}g\rho_{f}{}^{2}\left(T_{w}-T_{\infty }\right)x^{3}}{\mu_{f}{}^{2}}$ is the local Grashof number, and $Ec=\frac{u_{e}{}^{2}}{C_{p}\left(T_{w}-T_{\infty }\right)}$ is the Eckert number.

## 4. Physical Parameters

### 4.1. Displacement Thickness

The displacement thickness is written as

(15) $$\delta^{*}=\int_{0}^{\infty }\left(1-\frac{u}{u_{\infty }}\right)dy$$

By using Equation (11), it is written as

(16) δ∗=xRex−12∫0∞1−f′dη

### 4.2. Momentum Thickness

Momentum thickness is described as

(17) $$\delta^{**}=\int_{0}^{\infty }\frac{u}{u_{\infty }}\left(1-\frac{u}{u_{\infty }}\right)dy$$

By using Equation (11), it is illustrated as

(18) δ∗∗=xRex−12∫0∞f′1−f′dη

### 4.3. Skin Friction Coefficient

The skin friction coefficient is defined as

(19) $$C_{f}=\frac{2\tau_{w}}{\rho u_{e}{}^{2}}$$

After applying Equation (11), we get

(20) Cf=2Rex¯−12μnfμff″0e−Aθ0

### 4.4. Nusselt Number

Nusselt number is written in the following form

(21) $$Nu_{x}=\frac{hx}{k}$$

Here h is a convective heat transfer coefficient. In view of Equation (11), Equation (21) is shaped as

(22) Nux=−Rex¯12knfkfθ′0eBθ0

## 5. Solution Technique

The solution of Equations (12) and (13), with respect to Equation (14), is obtained by using the RK method. The method is executed in the following manner:

Let $f=F_{1}$, $\theta =G_{1}$ and convert Equations (14) and (15) into the system of first-order differential equations as

(23) $$\left.\begin{array}{l} {F_{1}}^{\prime }=F_{2}\\ {F_{2}}^{\prime }=F_{3}\\ {F_{3}}^{\prime }=AF_{3}G_{2}+\frac{\frac{\rho_{nf}}{\rho_{f}}\left[mF_{2}{}^{2}-\left(\frac{m+1}{2}\right)F_{1}F_{3}\right]-m-\omega \frac{{\left(\rho \beta \right)}_{nf}}{{\left(\rho \beta \right)}_{f}}\sin\left(\frac{\Omega }{2}\right)G_{1}}{\frac{\mu_{nf}}{\mu_{bf}}e^{-AG_{1}}}\\ {G_{1}}^{\prime }=G_{2}\\ {G_{2}}^{\prime }=-BG_{2}{}^{2}-\frac{\left(\mathrm{Pr}\frac{{\left(\rho C_{p}\right)}_{nf}}{{\left(\rho C_{p}\right)}_{bf}}\left(\frac{m+1}{2}\right)F_{1}G_{2}+\mathrm{Pr}.Ec.\frac{\mu_{nf}}{\mu_{bf}}F_{3}{}^{2}\right)}{\frac{k_{nf}}{k_{f}}e^{BG_{1}}} \end{array}\right\}$$

along boundary conditions

(24) $$\left.\begin{array}{l} F_{1}\left(0\right)=0,\\ F_{2}\left(0\right)=-\lambda ,\\ F_{3}\left(0\right)=\Omega_{1}\\ G_{1}\left(0\right)=1\\ G_{2}\left(0\right)=\Omega_{2} \end{array}\right\}$$

Here $\Omega_{1}$ and $\Omega_{2}$ are unknown boundary conditions.

To evaluate the accuracy of the results, the values of $f^{\prime \prime }\left(0\right)$ and $-\theta^{\prime }\left(0\right)$ against parameters β and Pr are compared with existing limited results [44,45] in Table 5 and Table 6.

The velocity and temperature distribution in the numeric form are displayed in Table 7 at ϕ=0.025%.

## 6. Result and Discussion

In this portion, obtained results in the form of velocity and temperature profiles, boundary layers parameters, skin friction, and Nusselt number for the flow of four different nanofluids: SiO_2_/DIW, Al_2_O_3_/DIW, PEG-GnP/DIW and PEG-TGr/DIW over wedge and plate are presented graphically. To view the influences of different nanoparticle volume fractions on a variety of results, fixed the values of mainstream velocity $u_{w}=0.01$, free stream velocity $u_{\infty }=0.04$ angle Ω=π/6, local Reynold number Rex=47615.9x, local Grashof $Gr_{x}=7.085\times 10^{8}x^{3}$ and Prandtl Number Pr=5.59576, and Eckert number $Ec=1.914\times 10^{-8}$. Other parameters are varied according to different nanoparticle volume fractions and are listed in Table 8.

### 6.1. Velocity Profiles

**Figure 20:**
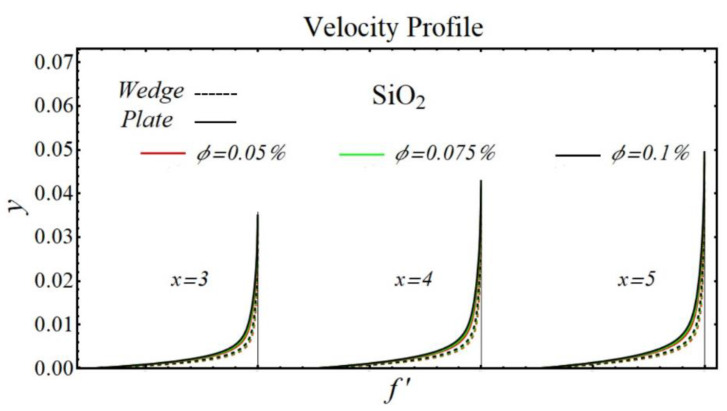
Velocity profile of SiO_2_/DIW nanofluids over the wedge and plate under the impact of nanoparticle volume fraction.

**Figure 21:**
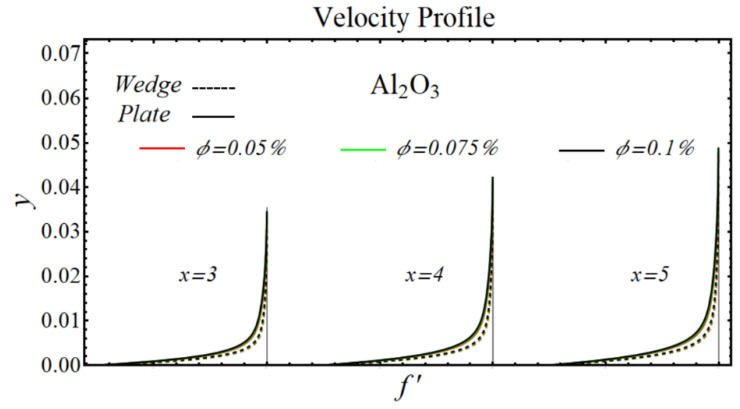
Velocity profile of Al_2_O_3_/DIW nanofluids over the wedge and plate under the impact of nanoparticle volume fraction.

**Figure 22:**
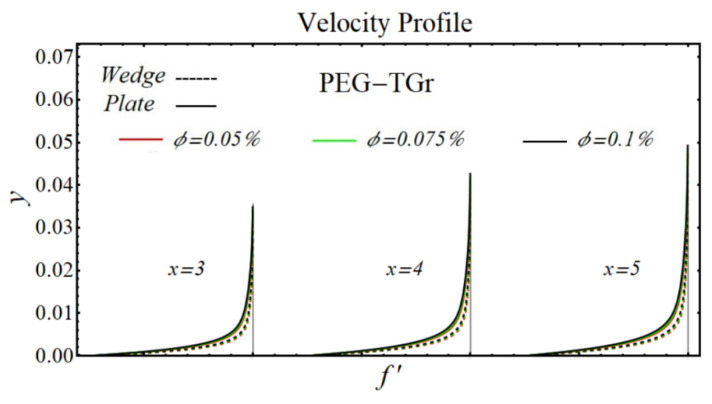
Velocity profile of PEG-TGr/DIW -nanofluids over the wedge and plate under the impact of nanoparticle volume fraction.

**Figure 23:**
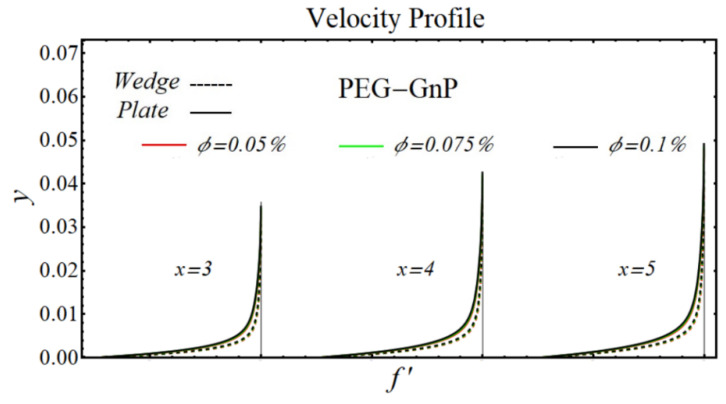
Velocity profile of PEG-GnP/DIW -nanofluids over the wedge and plate under the impact of nanoparticle volume fraction.

Figure 20, Figure 21, Figure 22 and Figure 23 illustrate the graphs of velocity profiles for SiO_2_/DIW, Al_2_O_3_/DIW, PEG-GnP/DIW, and PEG-TGr/DIW nanofluids under the influence of different nanoparticle volume fractions for both moving wedge and plate. It is seen that the values of viscosity of schematic nanofluids are amplified due to increasing the nanoparticle concentration. Given this evidence, the velocity profile of all schematic nanofluids over both geometries is decreased by raising the nanoparticle volume fraction. It is also observed that the profile of velocity is slowed down over a moving plate as compared to a moving wedge.

### 6.2. Temperature Profiles

**Figure 24:**
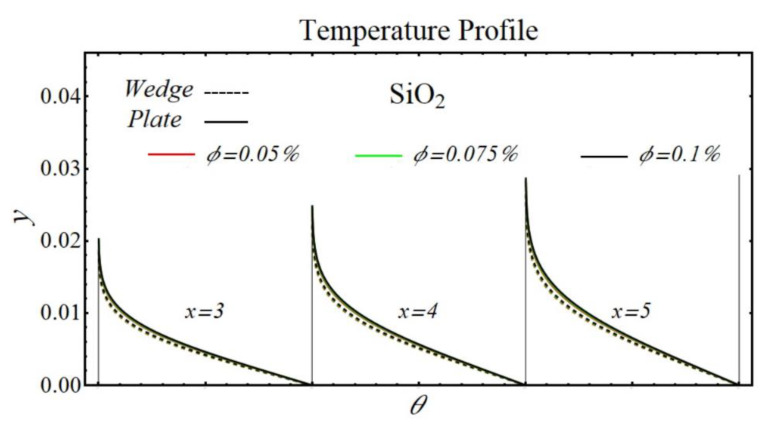
The temperature profile of SiO_2_/DIW nanofluids over the wedge and plate under the impact of nanoparticle volume fraction.

**Figure 25:**
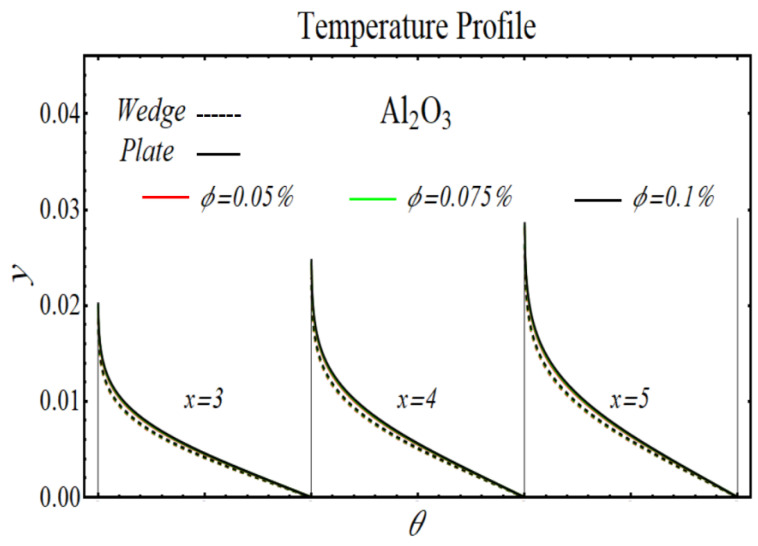
The temperature profile of Al_2_O_3_/DIW nanofluids over the wedge and plate under the impact of nanoparticle volume fraction.

**Figure 26:**
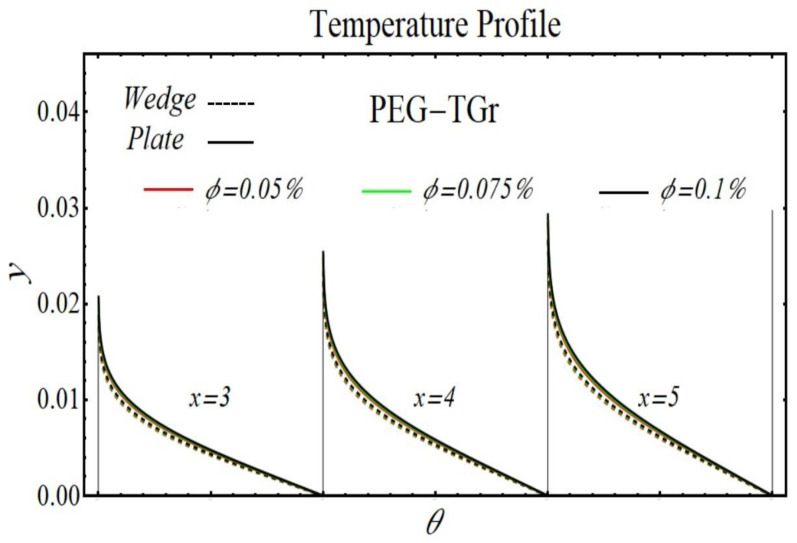
The temperature profile of PEG-TGr/DIW nanofluids over the wedge and plate under the impact of nanoparticle volume fraction.

**Figure 27:**
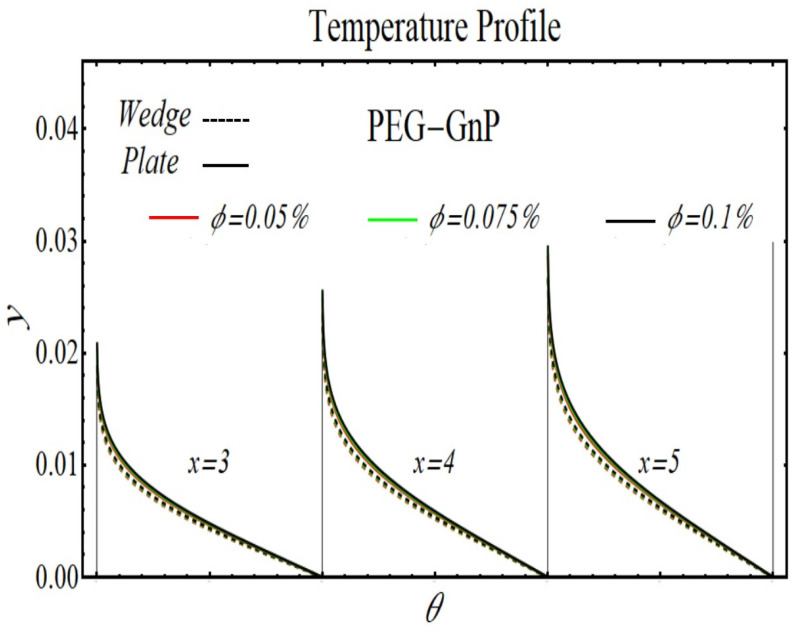
The temperature profile of PEG-GnP/DIW nanofluids over the wedge and plate under the impact of nanoparticle volume fraction.

Figure 24, Figure 25, Figure 26 and Figure 27 display the results of temperature profiles for all schematic nanofluids under the impact of different nanoparticle volume fractions over both geometries. It is detected from Figs. that the thermal conductivity is increased while specific heat is declined by increasing the nanoparticles concentration. In respect of changes in these properties, the distribution of temperature is increased. The prominent effects on temperature profile are found for PEG-GnP/DIW as compared to other nanofluids. Additionally, it is also seen that the temperature distribution is more raised in the case of a moving plate as compared to a moving wedge.

### 6.3. Physical Parameters

Table 9, Table 10, Table 11 and Table 12 dictate the results of boundary layer parameters such as momentum and thermal boundary region’s thicknesses, displacement thickness, and momentum thickness whereas the values of coefficient of skin friction and Nusselt number are illustrated in Table 13 and Table 14 for both geometries.

The results of momentum and thermal boundary region’s thicknesses of schematic nanofluids are computed numerically at a distinct location on the x−axis under the impact of nanoparticle volume fraction are listed in Table 9 and Table 10. It is seen that the thicknesses of momentum and thermal boundary region are enlarged by increasing the nanoparticle concentration and further increased along the parallel distance of the geometry’s wall. It is also seen that the momentum boundary layer thickness is greater than the thermal boundary layer thickness due to the dominant effects of viscosity as thermal diffusion. In addition, the boundary layer phenomena are produced more effectively on a plate as compared to a wedge.

In Table 11 and Table 12, the values of displacement and momentum thicknesses of schematic nanofluids are obtained under the impact of nanoparticle concentration at distinct positions on the x−axis. The value of displacement thickness shows the reduction in mass flow rate whereas the value of momentum thickness illustrates the reduction in momentum flow rate in the boundary layer region. The value of displacement thickness is raised by enhancing nanoparticle concentration and also increased along the parallel distance of the wall. It is also seen that the values of displacement thickness for flow over the plate are found greater than the values for flow over the wedge. Similarly, the momentum thickness is increased by raising of nanoparticle volume fraction as seen in Table 12. Moreover, it is observed that momentum thickness is found higher in the case of flow over the plate as compared to wedge.

Table 13 and Table 14 dictate the results of Nusselt number and coefficient of Skin friction for said nanofluids under the impact of nanoparticle concentration at a distinct location on the x−axis. The results demonstrate that the values of Nusselt number are enhanced by raising nanoparticle concentration due to the enhancement of thermal conductivity. Furthermore, the values of Nusselt number are also enhanced away from the origin along the x−axis. The values of Nusselt number are found higher when fluid flow over a wedge as compared to a plate. The coefficient of Skin friction is increased by raising nanoparticle concentration due to enhancement of viscous effects and is decreased along x−axis. The values of the coefficient of Skin friction are found larger in the case of flow over wedge as compared to the plate.

## 7. Conclusions

In the current investigation, the mathematical model for thermophysical properties of nanofluids is developed with help of experimental data and then used in transport equations to explore the boundary layer flow over plate and wedge. The results are obtained in the form of velocity and temperature and are further used to obtain the values of physical parameters. From the results, the following conclusions are exposed:

- The velocity is reduced whereas the temperature is enlarged due to amplifying viscosity and thermal conductivity respectively by variation of nanoparticle volume fraction for both wedge and plate.
- The velocity and temperature are more reduced and raised respectively in the case of flow over the plate as compared to a wedge.
- The momentum and thermal boundary layers are increased by enhancing nanoparticle volume but are found maximum in case of flow over a plate.
- The displacement and momentum thicknesses have followed the pattern of boundary layer thicknesses and are enhanced by variations in nanoparticle volume fraction.
- The skin friction coefficient and Nusselt number are raised with an enhancement in nanoparticles volume fraction but the maximum is found in the case of a wedge as compared to a plate.

## Figures and Tables

**Table 1:** The values of curve fitting parameters of Equation (2).

$\phi \left(\%\right)\to$	SiO_2_/DIW		Al_2_O_3_/DIW		PEG-TGr/DIW		PEG-GnP/DIW	
	0.025	0.050	0.025	0.050	0.025	0.050	0.0250	0.050
$\mu_{nf}$	0.8362	0.8457	0.8388	0.8564	0.8349	0.8496	0.8423	0.8521
n	1.0076	1.0070	1.0040	1.0025	1.0067	1.0067	1.0025	1.0024
$C_{1}$	0.01931	0.01902	0.0192	0.0191	0.0191	0.0186	0.0187	0.0184

**Table 2:** The values of coefficients of Equation (4).

	SiO_2_			Al_2_O_3_			PEG-TGr			PEG-GnP		
	a	b	c	a	b	c	a	b	c	a	b	c
$\mu_{nf}$	0.83	27.8	18,608	0.82	81.4	−27,636	0.83	35.0	19,260	0.83	32.2	12,840
n	1.00	0	0	1.00	0	0	1.00	0	0	1.00	0	0
$C_{1}$	0.10	−18.1	0.099	0.10	−11.1	0.10	0.10	−14.6	0.10	0.90	9.92	0.09

**Table 3:** The values of curve fitting parameters in Equation (4).

$\phi \left(\%\right)\to$	SiO_2_			Al_2_O_3_			PEG-TGr			PEG-GnP		
	0.025	0.050	0.075	0.025	0.050	0.075	0.025	0.050	0.075	0.025	0.050	0.075
$k_{nf}$	0.612	0.621	0.628	0.599	0.609	0.617	0.673	0.693	0.712	0.678	0.698	0.718
$10\times C_{2}$	0.049	0.051	0.052	0.044	0.045	0.046	0.058	0.006	0.062	0.058	0.059	0.061

**Table 4:** The values of coefficients of Equation (5) for density and heat capacity.

	SiO_2_		Al_2_O_3_		PEG-TGr		PEG-GnP	
	ρ	$C_{p}$	ρ	$C_{p}$	ρ	$C_{p}$	ρ	$C_{p}$
a	998.4	3.9945	1001.5	4.10735	991.5	4.066	991.4	4.066
b	2000	−19.2	2000	−4.52	2000	−4	2000	−4

**Table 5:** Comparison of results for $f^{\prime \prime }\left(0\right)$ with numerical results in [44] when ϕ=0 and n=1.

β	Present	[44]
0	0.46961	0.4696
1	0.92773	0.9277
2	1.23262	1.2326

**Table 6:** Comparison of results for $-\theta^{\prime }\left(0\right)$ with numerical results in [45] when ϕ=0 and β=1.

Pr	Present	[45]
1	0.57052	0.5705
2	0.74370	0.7437
6	1.11471	1.1147

**Table 7:** Numeric values of velocity and temperature distribution at ϕ=0.025%.

	SiO_2_/DIW		Al_2_O_3_/DIW		PEG-TGr/DIW		PEG-GnP/DIW	
η	$f^{\prime }$	θ	$f^{\prime }$	θ	$f^{\prime }$	θ	$f^{\prime }$	θ
0	0.25	1	0.25	1	0.25	1	0.25	1
0.3	0.8614	0.6560	0.8624	0.6558	0.8569	0.6644	0.8522	0.6646
0.6	0.9562	0.3573	0.9565	0.3578	0.9450	0.3690	0.9526	0.3693
1	0.9811	0.1122	0.9812	0.1133	0.9809	0.1202	0.9798	0.1204
2	0.9953	0.0009	0.9952	0.0010	0.9952	0.0011	0.9950	0.0011
4	0.9997	0	0.9997	0	0.9997	0	0.9997	0
6	0.9999	0	0.9999	0	0.9999	0	0.9999	0
8	1	0	1	0	1	0	1	0

**Table 8:** The values of parameters A and B at different nanoparticle volume fractions.

$\phi \left(\%\right)\to$	SiO_2_/DIW			Al_2_O_3_/DIW			PEG-TGr/DIW			PEG-GnP/DIW		
	0.025	0.050	0.075	0.025	0.050	0.075	0.025	0.050	0.075	0.025	0.050	0.075
A	3.87	3.78	3.69	3.87	3.81	3.75	3.82	3.75	3.67	3.75	3.70	3.65
B	0.098	0.101	0.105	0.088	0.090	0.092	0.116	0.120	0.124	0.116	0.119	0.123

**Table 9:** Momentum boundary layer thickness $10\times \delta_{M}$ of schematic nanofluids over the wedge and plate at different nanoparticle volume fractions.

		SiO_2_/DIW			Al_2_O_3_/DIW			PEG-TGr/DIW			PEG-GnP/DIW		
	$\begin{array}{c} \phi \left(\%\right)\to \\ \underset{↓}{x} \end{array}$	0.050	0.075	0.100	0.050	0.075	0.100	0.050	0.075	0.100	0.050	0.075	0.100
	2	0.277	0.292	0.308	0.277	0.290	0.302	0.277	0.291	0.306	0.281	0.292	0.305
**Wedge**	3	0.339	0.358	0.377	0.340	0.355	0.370	0.339	0.356	0.374	0.344	0.358	0.373
	4	0.392	0.413	0.435	0.392	0.410	0.427	0.392	0.411	0.433	0.398	0.414	0.431
	2	0.334	0.342	0.349	0.334	0.340	0.344	0.334	0.341	0.348	0.338	0.342	0.347
**Plate**	3	0.409	0.418	0.429	0.410	0.416	0.422	0.409	0.417	0.426	0.414	0.419	0.426
	4	0.472	0.483	0.495	0.473	0.480	0.487	0.472	0.482	0.493	0.478	0.484	0.491

**Table 10:** The thermal boundary layer thickness $10\times \delta_{T}$ of schematic nanofluids over the wedge and plate at different nanoparticle volume fractions.

		SiO_2_/DIW			Al_2_O_3_/DIW			PEG-TGr/DIW			PEG-GnP/DIW		
	$\begin{array}{c} \phi \left(\%\right)\to \\ \underset{↓}{x} \end{array}$	0.050	0.075	0.100	0.050	0.075	0.100	0.050	0.075	0.100	0.050	0.075	0.100
	2	0.182	0.184	0.186	0.182	0.184	0.185	0.185	0.188	0.191	0.186	0.189	0.192
**Wedge**	3	0.223	0.225	0.228	0.223	0.226	0.228	0.227	0.231	0.233	0.228	0.232	0.235
	4	0.258	0.261	0.263	0.258	0.260	0.263	0.262	0.266	0.269	0.263	0.268	0.271
	2	0.198	0.200	0.202	0.198	0.200	0.202	0.201	0.200	0.207	0.202	0.206	0.208
**Plate**	3	0.243	0.245	0.248	0.243	0.245	0.247	0.242	0.247	0.253	0.248	0.252	0.255
	4	0.182	0.184	0.186	0.182	0.184	0.185	0.185	0.188	0.191	0.186	0.189	0.192

**Table 11:** Displacement thickness $10\times \delta^{*}$ of schematic nanofluids over the wedge and plate at different nanoparticle volume fractions.

		SiO_2_/DIW			Al_2_O_3_/DIW			PEG-TGr/DIW			PEG-GnP/DIW		
	$\begin{array}{c} \phi \left(\%\right)\to \\ \underset{↓}{x} \end{array}$	0.050	0.075	0.100	0.050	0.075	0.100	0.050	0.075	0.100	0.050	0.075	0.100
	2	0.014	0.015	0.016	0.014	0.015	0.016	0.014	0.015	0.016	0.014	0.015	0.016
**Wedge**	3	0.017	0.018	0.020	0.017	0.018	0.019	0.017	0.018	0.020	0.018	0.019	0.019
	4	0.020	0.021	0.023	0.020	0.021	0.022	0.020	0.021	0.023	0.020	0.021	0.022
	2	0.020	0.021	0.023	0.020	0.021	0.023	0.020	0.021	0.023	0.021	0.022	0.023
**Plate**	3	0.025	0.026	0.028	0.024	0.025	0.026	0.025	0.026	0.028	0.026	0.027	0.027
	4	0.028	0.030	0.032	0.028	0.029	0.030	0.029	0.030	0.032	0.030	0.031	0.032

**Table 12:** Momentum thickness $10\times \delta^{*}{}^{*}$ of schematic nanofluids over the wedge and plate at different nanoparticle volume fractions.

		SiO_2_/DIW			Al_2_O_3_/DIW			PEG-TGr/DIW			PEG-GnP/DIW		
	$\begin{array}{c} \phi \left(\%\right)\to \\ \underset{↓}{x} \end{array}$	0.050	0.075	0.100	0.050	0.075	0.100	0.050	0.075	0.100	0.050	0.075	0.100
	2	0.009	0.010	0.011	0.009	0.010	0.011	0.009	0.010	0.011	0.001	0.010	0.011
**Wedge**	3	0.012	0.013	0.014	0.011	0.012	0.013	0.012	0.013	0.014	0.012	0.013	0.013
	4	0.014	0.015	0.016	0.013	0.014	0.015	0.013	0.014	0.015	0.014	0.014	0.015
	2	0.014	0.015	0.016	0.013	0.014	0.015	0.014	0.015	0.016	0.014	0.015	0.016
**Plate**	3	0.017	0.018	0.019	0.016	0.017	0.018	0.017	0.018	0.019	0.017	0.018	0.019
	4	0.009	0.010	0.011	0.009	0.010	0.011	0.009	0.010	0.011	0.01	0.010	0.011

**Table 13:** Nusselt number $Nu_{x}$ of schematic nanofluids over the wedge and plate at different nanoparticle volume fractions and distinct locations.

		SiO_2_/DIW			Al_2_O_3_/DIW			PEG-TGr/DIW			PEG-GnP/DIW		
	$\begin{array}{c} \phi \left(\%\right)\to \\ \underset{↓}{x} \end{array}$	0.050	0.075	0.100	0.050	0.075	0.100	0.050	0.075	0.100	0.050	0.075	0.100
	2	282.8	283.5	284.3	282.2	283.7	285.2	285.9	289.9	293.1	286.0	290.2	294.0
**Wedge**	3	346.3	347.2	348.2	345.6	347.4	349.3	350.2	355.1	359.0	350.3	355.4	360.1
	4	399.9	401.0	402.1	399.1	401.2	403.3	404.3	410.0	414.5	404.5	410.4	415.8
	2	254.4	258.5	259.1	254.4	258.7	260.0	260.7	264.3	267.1	260.7	264.4	267.9
**Plate**	3	311.6	316.6	317.3	311.6	316.8	317.4	319.3	323.7	328.1	319.3	323.9	328.1
	4	359.8	365.6	366.4	359.8	365.8	366.7	368.7	374.7	378.8	368.7	374.0	378.9

**Table 14:** Coefficient of Skin friction $10^{-3}\times C_{f}$ of schematic nanofluids over the wedge and plate at different nanoparticle volume fractions and distinct locations.

		SiO_2_/DIW			Al_2_O_3_/DIW			PEG-TGr/DIW			PEG-GnP/DIW		
	$\begin{array}{c} \phi \left(\%\right)\to \\ \underset{↓}{x} \end{array}$	0.050	0.075	0.100	0.050	0.075	0.100	0.050	0.075	0.100	0.050	0.075	0.100
	2	0.905	0.957	10.01	0.913	0.95	0.986	0.928	0.97	1.02	0.968	0.99	1.03
**Wedge**	3	0.739	0.781	0.827	0.745	0.776	0.805	0.758	0.792	0.829	0.79	0.814	0.84
	4	0.639	0.677	0.716	0.646	0.672	0.697	0.656	0.685	0.719	0.684	0.705	0.73
	2	0.631	0.699	0.741	0.667	0.695	0.722	0.677	0.707	0.742	0.707	0.728	0.751
**Plate**	3	0.515	0.571	0.604	0.545	0.568	0.589	0.553	0.578	0.606	0.577	0.594	0.613
	4	0.446	0.494	0.524	0.472	0.492	0.511	0.478	0.500	0.524	0.499	0.515	0.531

## Data Availability

Not applicable.
